# 

**Published:** 1894-12-15

**Authors:** 


**Notices of Births, Deaths, and Marriages** are inserted in this column at a charge of 2s. 6d. for an announcement not exceeding four lines.

**Notice to Advertisers and Subscribers.**

All Advertisements, Orders for Copies of The Hospital, and Business Communications should be addressed to **The Publisher, NOT TO THE EDITOR,** The Hospital, 428, Strand, W.C.

The Cost of Subscription, post free, is as follows:—Per week, 2½d.; per quarter, 2s. 8d.; per half-year, 5s. 3d.; per annum, 10s. 6d. Foreign:—Per Annum, 12s. 8d.; payable, in all cases, in advance.

### NOTICE TO CORRESPONDENTS.

All MS., Letters, Books for Review, and other matters intended for the Editor should be addressed The Editor, The Lodge, Porchester Square, London, W.

The Editor cannot undertake to return rejected MS., even when accompanied by stamped directed envelope.

**"Burdett's Hospital Annual," 1894.**

Fifth year of publication. 900 pp. Boards, gilt lettering. A most complete guide to British, Colonial, Indian, and American Hospitals, Dispensaries, Nursing and Convalescent Institutions, and Asylums. Price 5s. Published by The Scientific Press (Limited), 428, Strand, W.C.

**NOTICE.—The Index for Vol. XVI. (ending September 24th, 1894) is on sale at the Office of this Journal, Price 2½d., post free.**

### RADCLIFFE INFIRMARY, OXFORD.

### SCRAPS AND GLEANINGS.

A hospital is to be built for the lepers in Iceland.

A generous gift of £500 has been made by Miss Allingham to the National Lying-In Hospital, Holles Street.

According to an American medical journal, acute inflammatory rheumatism must be classed amongst the microbic diseases.

Opium advocates are triumphing in the statement that opium eaters, almost without exception, were exempt from the plague at Hong Kong.

The New Southport Infirmary is advancing steadily. The three principal wards are nearly complete, and the grounds are about to be laid out.

Baron Albert de Rothschild has given a large sum of money to establish a cancer ward for women at the Empress Elizabeth Hospital, Vienna.

Owing to pressure in the work of the Belfast Royal Hospital, an extra assistant attending and an extra assistant house surgeon are to be appointed

At the North Staffordshire Infirmary and Eye Hospital, the disinfector becoming out of order a new Washington Lyon apparatus has been adopted.

The Longton Cottage Hospital, Stafford, is doing a good work and flourishing, in spite of the fact that 23 manufactories remain off the list of subscribers.

A new fever wing, detached from the main building, is to be added to the Mater Misericordiæ Hospital, increased accommodation being urgently needed.

The Manchester Infirmary Board are considering the proposition to hold post graduate lectures in the wards during the months of June and July in each year

The effort is being made by the supporters of the Northern Counties Hospital for Incurables, at Mauldeth and Walmersley, to raise a sum of £4,000 towards an out-pension fund.

The Management of the Truro Dispensary are thinking of purchasing the premises they now occupy. The medical work and the finances of the institution are both satisfactory.

A scheme is being considered at the Dudley Guest Hospital whereby working men's representatives shall become eligible to join the management, and have a right to attend the meetings of the institution.

The proposition to establish an isolation hospital at Devizes is gaining ground. There is an apparent need of such an institution, seventy-four cases of diphtheria alone being reported in the district, with a mortality of 12 per cent.

Most vigorous measures are being taken to make the forthcoming bazaar in aid of the Derbyshire Royal Infirmary a success. If as much enthusiasm can be awakened as was shown at Southport, the institution will be very substantially benefited.

Professor Donath, of Budapest, has arrived at the discouraging conclusion that the human race is physically deteriorating rather than improving at the present time. He has formed this opinion after an examination of the statistics of European armies.

The Boston Hospital was shown to possess an adverse balance at the recently held annual meeting. Mr. Read, who was present, handed to the chairman a cheque for £181, the amount due, as a gift from a fund of which he was trustee, intended for the purpose of benefiting charities.

The new Consumption Hospital for Ireland, now in process of erection at Newtown-mountkennedy, is making satisfactory progress. The building will consist of three blocks, and peculiar care is being taken to make the sanitary and ventilating arrangements as perfect as possible.

The Manchester Royal Infirmary has been approached by the Commissioners of the Royal Hospital, Chelsea, on the question of admitting some of their pensioners when medical treatment appeared likely to restore them to health. The infirmary declined to consider the proposal.

Two hundred and forty-seven patients have been attended in their own homes during the month of November by the St. Patrick's Nurses, affiliated with Queen's Jubilee Institute, of whom 150 were new cases; 166 applications for attendance are reported, while the number of visits paid reaches 2,784.

Doctors in France are not allowed to supply their patients with medicine when a chemist exists within a prescribed distance. He may administer certain specific remedies necessary in urgent cases. He may only use any serum guaranteed by the proper authorities.

It is a pity that the Reigate and Redhill Cottage Hospital is not better supported. The institution fulfils a useful purpose and is most necessary, more accommodation being now required. Surely in a flourishing residential neighbourhood no very urgent appeal should be necessary to secure adequate support.

Monsieur Dubois is agitating for some reorganisation of hospital regulations in Paris. He has asked the municipal council to regulate the payment of patients in the hospitals; to supply statistics of paying patients from all hospitals, and the length of their sojourn therein; and to institute a fixed tariff for surgical operations.

Rather a characteristic example of the canny Scot was given at a hospital meeting at Edinburgh recently. A local band offered to give a Sunday concert for the infirmary. Some one present remarked it was a pity the infirmary had been asked. For the sake of avoiding possible offence, it was decided to decline the offer, but in a "general way."

A new sanatorium for the isolation of infectious cases other than small-pox is about to be erected at Crewe, the Corporation of that town having purchased five acres of land for the purpose. The sanatorium will be about two miles from the already existing small-pox hospital, which has been doing good work for some years past, and it is proposed to expend between £4,000 and £5,000 upon the new building.

### THE STUDENT OF MEDICINE.

### APPOINTMENTS.

Alfred Adams, M.A., M.B., B.Ch.Oxon, M.R.C.S., L.R.C.P., appointed House Physician to the Radcliffe Infirmary, Oxford. Dr. Dudgeon, appointed Medical Officer for the No. 8 (South Hackney) District of the Hackney Union. Percy James Edmunds, M.R.C.S., L.R.C.P., B.Sc.Lond., appointed District Medical Officer for St James, and Public Vaccinator for St. James and St. Anne, under the Guardians of the Westminster Union. J. Griffiths, M.A.Camb., M.B.Edin., appointed Assistant Surgeon to the Addenbrooke's Hospital, Cambridge. J. C. Heaven, L.R.C.P.Lond., M.R.C.S., reappointed Medical Officer of Health for the Keynsham Rural Sanitary District. H. Oswin Johnson, M.R.C.S.Eng., L.R.C.P.Lond., appointed Medical Officer and Public Vaccinator to the Parkes District of New South Wales; also Surgeon to the Parkes District. J. H. Joyce, B.A., M.B., B.C., appointed House Surgeon to the Kent and Canterbury Hospital. William Ernest Miles, F.R.C.S.Eng., appointed House Surgeon to the Radcliffe Infirmary, Oxford. E. V. Pegge, L.R.C.P.Lond., M.R.C.S., appointed Medical Officer for the Briton Ferry District of the Neath Union. W. E. Porter, M.D.Edin., D.P.H.Camb., appointed Public Vaccinator for the Wood Green District of the Edmonton Union. J. Nigel Stark, M.B., F.F.P.S.G., appointed Assistant Obstetric Physician to the Glasgow Maternity Hospital. Cecil E. Stephen, M.B., C.M.Edin., appointed Medical Officer of Health to the North Witchford Rural Sanitary District. H. B. Vincent, M.R.C.S.Eng., appointed Medical Officer of Health to the Erpingham Rural Sanitary District. Dr. Watts, appointed Medical Officer for the Ilchester District of the Yeovil Union. Samuel West, M.D., F.R.C.P.Edin., appointed Examiner in Medicine at the University of Oxford. John T. Wilson, M.B., C.M. Aberd., appointed Medical Officer of Health to the Lanark County Council.

### VACANCIES.

The following vacancies are announced this week, the amount of salary in each case being given after the name of the institution:

### Resident Medical Officers.

Kent and Canterbury Hospital.—Assistant House Surgeon; unmarried. Salary, £50 per annum, with board and lodging. Applications to the Secretary by December 29th.

North-Eastern Hospital for Children, Hackney Road, Shoreditch, N.E.—House Physician. Appointment for six months; at expiration of this term he will be required, if eligible, to serve as House Surgeon for further period of six months. Salary as House Physician at the rate of £60 per annum, and as House Surgeon at the rate of £80 per annum. Doubly qualified. Applications to the Secretary, at the City Offices, 27, Clement's Lane, Lombard Street, E.C., by December 18th.

Westminster General Dispensary, 9, Gerrard Street, Soho, W.—Honorary Surgeon and Resident Medical Officer. Applications to the Secretary by December 20th, at Ten o'clock.

Wolverhampton Eye Infirmary.—House Surgeon. Salary £60 per annum, with rooms, board, and washing. Applications to the Secretary by December 17th.

### Non-Resident Medical Officer.

Westminster Hospital. — Fourth Assistant Surgeon. Must be F.R.C.S.Eng. Candidates must transmit certificate of age, and attend the House Committee on January 1st.

### UNIVERSITIES AND COLLEGES.

### UNIVERSITY OF LONDON.

In the recent class list of the University of London for the M.B. Degree, the London Medical Schools are represented as below:

| | First Division. | Second Division. | Total. |
|---|---|---|---|
| Guy's | 5 | 7 | 12 |
| St. Bartholomew's | 3 | 8 | 11 |
| University College | 1 | 9 | 10 |
| St. Thomas's | 4 | 4 | 8 |
| St. Mary's | 3 | 5 | 8 |
| London School of Medicine for Women | 1 | 6 | 7 |
| London | 0 | 5 | 5 |
| Middlesex | 2 | 1 | 3 |
| King's College | 1 | 1 | 2 |
| St. George's<br>Westminster<br>Charing Cross | 0 | 0 | 0 |

Of the larger schools Guy's, and of the smaller ones St. Mary's. show the best record. The success of the Women's School speaks well for the clinical teaching at the Royal Free Hospital.

### SOCIETY OF APOTHECARIES OF LONDON.

The following candidates passed in:

SURGERY.—W. Allingham, St. George's Hospital; M. Carter, Charing Cross Hospital; R. W. S. Christmas, Charing Cross Hospital; E. E. Dufty, Sheffield; G. W. Gostling, University College Hospital; R. Jones, London Hospital; H. A. Julius, St. Thomas's Hospital; G. Lowsley, St.

**THE STUDENT OF MEDICINE**—*continued.*

Bartholomew's Hospital; R. W. Middleton, Leeds; H. W. Ramsay, St. Mary's Hospital; W. L. Roberts, St. Mary's Hospital; F. E. Saunders, St. Thomas's; G. A. Simpson, London Hospital; A. M. Thornett, Royal Free Hospital; E. A. Tudman, University College Hospital.

Medicine. Forensic Medicine, and Midwifery.—T. D. Bell, University College Hospital; A. K. Gordon, St. Mary's Hospital; G. W. Gostling, University College Hospital; A. W. Haines, Birmingham. T. S. F. Hudson, Birmingham; A. E. Lovett, London Hospital; W. E. Stanton, London Hospital; F. H. P. J. U. Walker, St. George's Hospital.

Medicine and Midwifery.—R. G. Worger, Guy's Hospital.

Medicine.—A. L. M. Churchill, Westminster Hospital.

Forensic Medicine and Midwifery.—W. Sutcliffe, Birmingham.

Midwifery.—F. W. Mawby, Guy's Hospital; A. M. Thornett, Royal Free Hospital.

To Messrs. Allingham, Carter, Christmas, Dufty, Gosling, Haines, Hudson, Jones, Julius, Mawby, Stanton, Tudman, Walker, and Miss Thornett was granted the diploma of the Society.

